# Highlighting Personality and Social Psychological Theories From Majority World Contexts: Introduction to the Special Issue

**DOI:** 10.1177/10888683251364486

**Published:** 2025-08-25

**Authors:** Stephen Baffour Adjei, Pegah Nejat, Amber Gayle Thalmayer, Jonathan M. Adler

**Affiliations:** 1 University of Oslo, Norway; 2 Akenten Appiah-Menka University of Skills Training and Entrepreneurial Development, Kumasi, Ghana; 3 Shahid Beheshti University, Tehran, Iran; 4 University of Zurich, Switzerland; 5 University of the Free State, South Africa; 6 Olin College of Engineering, Needham, MA, USA

**Keywords:** decolonizing knowledge, globalizing psychology, indigenous theories, majority world, social and personality psychology

## Abstract

Since the inception of scientific psychology in the 19th century, the lead in conceptualizing scientific phenomena has been taken by scholars in Western contexts (North America, Western Europe, Australia, and New Zealand), who comprise only 11% of the world’s population. Today, the science and practice of psychology continue to be dominated by Western theoretical perspectives. Recognizing the necessity for inclusive excellence in the field and the barriers that Majority World scholars face in joining the global knowledge economy, *Personality and Social Psychology Review* (PSPR) has taken several steps toward global inclusion. To further realize this goal, this Special Issue brings together nine contributions that reflect personality and social psychological theory rooted in diverse Majority World contexts, specifically stemming from African, Latin American, Middle Eastern, East and South Asian, and Indigenous American scholars. The contributions reflect several cross-cutting themes: the deeply historical contexts in which personality and social psychological phenomena play out in different geographies today; the important particularities of widely studied concepts in specific local contexts; and the dynamic interplay between individual people and the specificity of their social contexts. By curating indigenous concepts and theories, we aim to further catalyze dialogue across cultural distances in the field and to demonstrate how a decolonized editorial process can help promote inclusive science to improve the dominant perspectives in personality and social psychology.

## Introduction

In our increasingly interconnected society, global perspectives in psychological science and practice are needed more than ever. Without this broadened approach, we cannot adequately understand and address the myriad challenges of humanity. However, since the inception of scientific psychology in the 19th century, the power to theorize, conceptualize, and name psychological phenomena has been dominated by scholars in Western contexts (United States, Canada, Western Europe, Australia, and New Zealand; Oppong, 2024). Recent studies show that psychological science continues to be dominated by and rooted in the West, though it is home to only 11% of the world’s population (Krys et al., 2024; Thalmayer et al., 2021). To work toward making psychology a more complete science of the human experience, in this Special Issue, we highlight theories and constructs relevant to social and personality psychology from the “Majority World.” We use the term Majority World to describe non-Western societies in Africa, Asia, Latin America, and the Caribbean, where most humans live (Alam, 2008; Kagitcibasi, 2002).^1^

Majority World scholars face many barriers to conducting research. These include heavy teaching loads, high student numbers, the burden of writing in borrowed language, inadequate institutional research infrastructure, limited national investment in research, political and other social hurdles, and lack of academic leadership skills, funding, and opportunities for professional collaboration (Adjei et al., 2025; Uskul et al., 2024). Also, the dominant Western psychological knowledge base ignores the worldviews, needs, and epistemologies of people in other regions (Ratele, 2017; Suffla et al., 2023), with problematic consequences for psychological theory and applications in treatment, education, and policy (Christopher et al., 2014; Sodi & Bojuwoye, 2011). From Africa (Adjei et al., 2025; Ratele, 2017; Tchombe, 2022) to Asia and Latin America, psychological research and practice continue to privilege imported Western canons of knowledge rather than locally generated theories and concepts (Uskul et al., 2024). Two unfortunate consequences of these pervasive challenges are epistemic imitation and oppression (Adjei et al., 2025; Adjei, in press; Dotson, 2012). Epistemic imitation represents an unfair knowledge practice in which the teaching, research, and application of psychological knowledge in Majority World regions are predominantly oriented toward, centered around, and derived from Western knowledge systems and lived experiences as though they are culturally neutral, apolitical, and intrinsically liberating (Adjei, in press). The complementary phenomenon, epistemic oppression, involves the exclusion of epistemic agents as knowers that reduces their capacity to participate in knowledge production and results in deficiencies in social knowledge (Dotson, 2012). Both epistemic imitation and oppression can be likened to what Nobles (2013) refers to as “conceptual incarceration,” in which a knower or epistemic agent is given a set of predetermined concepts and definitions to use in the process of knowing, consequently inhibiting them from a deeper understanding of their own local realities.

Together, these practices block the multiple perspectives that are required to tackle global issues. This Western bias in research has been costly as it excludes phenomena of scientific, practical, and theoretical utility from the Majority World contexts (Adetula et al., 2022; Mughogho et al., 2023), limits the generalizability of findings, obscures identification of cross-cultural universals (Norenzayan & Heine, 2005; van de Vijver, 2013), and ultimately impedes our ability to generate a deeper and more complete understanding of cultural influences on psychological processes (Apicella et al., 2020).

## Systemic Disadvantages Faced by Majority World Scholars Within the Western Publishing Ecosystem

Psychology is alive and well around the world, with many regional societies, scholarly journals, and timely topics of interest (e.g., Uskul et al., 2024). We recognize that Majority World scholars are already contributing original knowledge to positively shape and expand extant theoretical conceptions in social and personality psychology. Indeed, it is important for Western scholars to appreciate that personality and social psychology are active fields in the Majority World, whether or not Western scholars are aware of that work. However, there are several reasons that the work of Majority World scholars too seldom appears in the most prestigious Western outlets, such as Personality and Social Psychology Review (PSPR). The reasons include an even higher, almost insurmountable, bar for success, lack of mentors with experience to help them navigate the norms, various forms of gatekeeping, and low representation of Majority World scholars in key editorial roles.

Majority World scholars who wish to publish in PSPR or similar Western journals may lack mentoring and developmental support from established scholars to help them navigate the norms of academic writing and publishing. Also, there are gatekeeping biases that are manifested when research from the Majority World and/or submitted by researchers from non-Western contexts is evaluated, such as outrightly dismissing content as irrelevant, authors “being asked to justify the study context and explain why it is a worthwhile topic to study, or to add a western comparison group” (Uskul et al., 2024, p. 11). Majority World scholars may also find themselves in a dilemma when they write about their research for Western journals: whether to stick to and write about their original and ecologically valid ideas in support of their epistemic agency, or to conform to the expectations of Western editors and their default knowledge structures to appear legible and get published (Adjei et al., 2025). For example, it is possible to study a construct like personality traits in Majority World contexts and to reproduce some Western findings, but this does not mean they are the optimal constructs for understanding people locally, or that they map onto local discourse, needs, or interests (e.g., Thalmayer et al., 2022). Of course, the demand to align one’s research output with the culture of journals in one’s field is a universal challenge scholars face, but the potential for misalignment and epistemic burden is much greater among Majority World scholars than their Western counterparts.

Another contributing factor to the persistent structural inequities and systemic bias is the under-representation of Global Majority scholars in key editorial roles such as editors, associate editors, editorial board members, and reviewers, compared to the overwhelming majority of Western scholars (e.g., over 90% of white scholars) occupying these roles in top psychology journals (Roberts et al., 2020). Western-centric practices and systemic disadvantages for Majority World scholars within the current Western publishing ecosystem require deliberate interventions at multiple levels (Buchanan et al., 2021) to achieve broader inclusion and diversity in our journals.

## Toward Broader Global Inclusion at PSPR

Anchored on the strong foundation of PSPR’s history, the editorial team that took office at the beginning of 2022 determined to steer the journal in a new direction. A direction that focused attention on “*what* we study, *who* has the opportunity to enter and eventually lead our field, and *why”* (Adler, 2022, p. 87). The paradigm shift at PSPR is not only borne out of the awareness of structural exclusion of many scholars from the Global Majority, but also the recognition that scholarly journals are constantly involved in “prophetic framing and frame shifting.” Editorial decisions and journals’ tables of contents continuously shape the landscape of academic publishing in personality and social psychology by determining the kinds of papers that are published, and authors who can and should publish their perspectives in certain outlets (Adler, 2022). Thus, the new direction calls for the field to elevate previously excluded perspectives on personality and social psychology. Multiple viewpoints are necessary to guide our field toward a more balanced and inclusive understanding of human psychology (Apicella et al., 2020) and to broaden our field’s awareness of the wonderful diversity of the human experience and the many types of questions we can ask about it.

In line with its new focus, the current PSPR editorial team has taken initial steps toward broader global inclusion in this journal. These include constituting an Editorial Board with more than one-third of scholars working outside Western nations, developing an Emerging Editor Board with broad global representation, and initiating an Editorial Fellowship program to foster pathways to editorial leadership for non-Western scholars. This Special Issue is a further step to concretize the core values and mark a new chapter in the history of PSPR. The goal of this Special Issue is to highlight personality and social psychological theories from Majority World contexts in order to catalyze dialogue between Western and Majority World scholars, and among Majority World scholars in the field. Specifically, the contributions in the Special Issue address two important questions: (a) How can social and personality psychological concepts and theories from Majority World contexts contribute to a truly global science of personality and social psychology? and (b) What can dominant Western psychology learn from social and personality psychological concepts and theories rooted in and oriented around perspectives from Majority World contexts?

Two critical considerations guided how we addressed these key questions. First, we sought to align the core editorial values and processes of the Special Issue with its content as a mechanism to ‘decolonize’ not only the content on our pages, but also the ways in which this issue came to be. To decolonize in this sense is to challenge the prevailing assumptions about human behavior that are rooted in Western knowledge frameworks (Bryant, 2024), to disrupt systems of oppression and marginalization in psychological research (Adjei, in press; Bryant, 2024), and to expand upon Western-centric ways of thinking by centering indigenous knowledge (Mitova, 2020). Second, we sought to use the editorial process itself to offer opportunities that promote epistemic agency among marginalized scholars. We hope that these two considerations together would help foster an inclusive publication ecosystem and catalyze meaningful and mutually beneficial dialogue among Majority World scholars and between Majority World and Western scholars.

The focus of this Special Issue aligns with Special Issues and Thematic Sections at other journals devoted to the theme of decolonizing and globalizing psychological science (e.g., Adams et al., 2015; Awad et al., 2024; Readsura Decolonial Editorial Collective, 2022; Santana et al., 2025). Though our primary goal is to highlight indigenous social and personality psychological theories and concepts from Majority World contexts, ultimately, we hope that this Special Issue will contribute to the broader discussion on decolonizing psychological science in practice. We aim to reach at least three audiences: Western readers of PSPR oriented around Western knowledge structures; Majority World readers and scholars who apply Western theoretical approaches to the study of social and personality psychology; and readers from both Western and Majority World contexts who utilize indigenous and decolonial theoretical perspectives to understanding social and personality psychological phenomena. We believe that this Special Issue is one such concrete step to build *epistemological bridges* (Adjei, in press) that help us move away from *universality* to *pluriversality*—emphasizing the multiple, diverse, and interconnected ways of understanding the world around us (Mignolo, 2018). Re-imagining science and knowledge as pluriversal can expand the prevailing limited theoretical perspectives in social and personality psychology.

## Decolonizing the Editorial Space: Aligning Core Editorial Values and Process With Content

Consistent with our goal to elevate theoretical and conceptual issues from Majority World contexts, the papers in this Special Issue comprise a carefully curated set collected in response to an open call for submissions. In seeking to reach scholars who might not otherwise learn about initiatives at PSPR, we made proactive outreach to more than 80 psychological organizations and thought leaders in all regions of the world, alerting them to the call for submissions. Globalizing efforts require this kind of proactive outreach to reach the scholars whom we wish to invite into the conversation in Western journals. The final set of accepted manuscripts represent contributions from a range of scholars (we prioritized scholars from and working in Majority World, but also included some scholars from diaspora communities and minoritized groups from Western contexts), broad geographic regions (Africa, Latin America, Middle East, East and South Asia, and some Indigenous Western scholars), variety of levels of analysis (individual, dyads, small groups, and societal), and grounded in both social and personality psychology. Mindful of the power differentials in theorizing in psychology and the systemic exclusion of the many original, mature, and thriving voices in the Majority World, we intentionally left the content areas within personality and social psychology in the call for proposals open and unconstrained. Instead, we emphasized submissions that engage with original conceptual and theoretical perspectives and experiences of people outside Western contexts.

Rather than a rehash of dominant topics in Western psychology, the content of the set in this Special Issue covers a range of relevant topics that center experiences of communities in Majority World contexts. The contributions draw from, emphasize, and integrate local scholarship and context-specific indigenous understandings from a broad geographic distribution. We believe that, by drawing from, emphasizing, and integrating local scholarship, the Special Issue not only provides a purposeful and supportive space for elevating specific contributions, but it also calls significant attention to historically excluded scholarship and theories that Western scholars might not otherwise encounter or engage with without actively pushing beyond their usual sources. The encouragement to cite local journals and authors in their contributions to this Special Issue broadens the visibility and impact of local scholarship and ultimately contributes to the continuous effort for a lasting transformative effect on theoretical perspectives that dominate our field.

This Special Issue also sought to intervene in dominant Western editorial processes in terms of shifting from a primarily transactional approach to a more relational one (e.g., Adler & Singer, 2023). Traditional editorial models typically operate with a hierarchical approach in which members of the editorial team make individual decisions, but the final call rests with the editor. In contrast, we opted for a more horizontal editorial leadership structure among the editorial team. The idea for this Special issue grew out of a discussion at the annual event the journal holds for its full editorial team, including the Editorial Board and Emerging Editor Board, and members of both Boards have served as reviewers for every manuscript. In addition, the four co-editors of this Special Issue represent two Editorial Fellows, one Associate Editor, and one Editor. All members of the editorial team were invited to reflective, open, and frank discussions at collaborative editorial meetings, where we decidedly embraced the principles of decolonial work in our editorial approach.

We also moved away from the more transactional editorial approach where editors work with authors mainly via e-mail communications and make editorial decisions based on traditional Western editorial standards and practices, toward a more relational approach that allowed for correspondence and meetings between authors and action editors alongside the formal submission and evaluation process (Adler & Singer, 2023). While this relational decolonial approach can be labor intensive, it is rewarding and liberating both for authors who face challenges writing in borrowed languages for Western psychology journals and for editors from Western contexts who are interested in disrupting and fostering a genuine, actionable change in the existing taken-for-granted colonial editorial structure. The relational editing approach we adopted is a demonstration of our commitment to decolonizing both the content and process of knowledge production in psychology. Throughout the process, we focused on honoring the epistemic agency of Majority World scholars —that they should be treated as having authority in their own existential experiences and the power to name subjectivities in psychology, and to conceptualize from their own indigenous cultural experiences (Adjei, in press; Adjei et al., 2025). We thus reflexively and continuously reminded ourselves during the editorial process not to develop authors’ contributions into narrow Western approaches—that the final papers from authors needed to be *legible* to both Majority World and Western audiences, but we were mindful not to necessarily force conformity to Western conventions. Special issues afford editorial teams an opportunity to experiment with special editorial practices, which might inform advocacy for broader and more enduring structural change.

## The Contributions to this Special Issue

Our call for papers for this special issue was deliberately broad and content-agnostic (other than featuring personality and social psychology). The authors in this issue bring perspectives from East Asia (China, Hong Kong, South Korea), Ethiopia, India, Iran, Latin America (Brazil, Chile, Venezuela), and Indigenous North America. We see three cross-cutting themes emerging from the papers collected here.

First, several papers demonstrate the ways in which the forces of history are instantiated in everyday life. For example, the landscape of prejudice in contemporary Ethiopia cannot be understood outside the shifting political history of the country over centuries, and especially in the past 50 years (Tessema et al., 2025). The specific manifestation of authenticity in China, which takes a “round outside and square inside” shape, represents an inheritance across millennia of Confucianism (Du et al., 2025). And the concept of Mino-Bimaadiziwin, the good life, which flourishes today as the result of careful cultivation across generations of Anishinaabe Native American ancestors (Nelson et al., 2025). As a set, these papers demonstrate the deeply historical contexts in which personality and social psychological phenomena play out in different geographies today.

Second, several papers demonstrate the ways in which dominant Western approaches to understanding psychological phenomena fall short in capturing those phenomena in Majority World contexts and offer alternative visions that emerge from the local contexts. For example, ideals of relationship quality look very different in East Asia and require us to understand them through the lens of the East Asian indigenous philosophical tradition of naïve dialecticism that values contradictions, embraces change, and emphasizes interconnectedness (Joo & Lam, 2025). Western approaches to understanding workplace gender equity interventions don’t adequately explain how they operate in Latin American contexts, thus necessitating the need for a decolonial approach (Oliveira-Silva et al., 2025). Efforts aimed at demarginalizing communities cannot adopt universal approaches, but must embrace the distinctive intersectionality of their local contexts, as demonstrated through caste-based marginalization in India (Singh et al., 2025). And, while the notion of honor is recognized universally, it varies regionally in frequency, chronicity, and intensity according to the Costly Morality Theory of Honor (Karimi-Malekabadi & Oyserman, 2025). As a set, these papers demonstrate the important particularities of widely studied concepts in specific local contexts.

Third, several papers focus on a very specific phenomenon within a very particular local context. For example, the Brazilian notion of Jeitinho, or “little way,” captures a specific strategy of getting things done (Pilati & Fischer, 2025). The construct of “lying flat” in China and Hong Kong captures the experiences of young people who refuse to chase status and success (Hui et al., 2025). And the Native American concept of Mino-Bimaadiziwin, which captures a specific understanding of the good life (Nelson et al., 2025). Individually, each of these papers brings to life a phenomenon that may be unfamiliar to readers outside of these specific local contexts, but which reveals the kaleidoscopic diversity of the human experience. As a set, the papers that adopt this approach help reveal the dynamic interplay between individual people and the specificity of their social contexts.

Across all contributions to this Special Issue, the authors authoritatively draw from their indigenous theoretical approaches and concepts to name subjectivities and to center theoretical traditions from local communities as valid knowledge authored by legitimate knowers. These amplified voices from underrepresented regions of the world both challenge and enrich our understanding of social and personality psychological phenomena. By embracing and acknowledging the epistemic authority of authors, we sought to demonstrate how a decolonized editorial process can denaturalize Western normativity, expand Western journals’ capacity to name and conceptualize from context-sensitive ideas, and valorize lived realities and indigenous knowledge systems from Majority World settings. The contributions weave together into a rich tapestry of theoretical and conceptual social and personality psychological perspectives, offering alternative theoretical narratives for both Majority and Western scholars to engage with.

## Conclusion: Amplifying Majority World Perspectives to Enhance the Diversity of Global Treasury of Knowledge

The promise of the science of social and personality psychology in understanding and improving the human condition demands that we create equitable spaces for multiple ontological and epistemological perspectives from scholars and practitioners located in diverse contexts to be said and heard. Transforming the colonial infrastructure of the publishing ecosystem to remedy the historically biased corpus of Western psychological insight requires consistent, purposeful, and actionable inclusive interventions that offer empowering spaces for global majority scholars to think from their own epistemic locations and within the imaginaries of their indigenous knowledge systems. This Special Issue has attempted to create a visible space for a kaleidoscopic view from Majority World contexts to engage with conceptual and theoretical perspectives that speak to context-sensitive experiences of people outside Western contexts. However, we do acknowledge that it is impossible to be comprehensive in including Majority World perspectives in a single Special Issue.

In addition to sharing their research in other outlets, we hope this Special Issue has provided another essential and powerful platform for Majority World scholars to participate in knowledge production and share their experiences as legitimate knowers in one of the leading Western journals in social and personality psychology. While this Special Issue is not the ultimate solution to radical shifting of the publication system, we see it as an important invitation for more work like this to appear in PSPR and other social and personality psychology journals, but there is a lot of work to do to make that possible. Welcoming new voices into PSPR and other impactful Western psychology journals is only the first step. The success of this Special Issue in catalyzing dialogue with dominant Western approaches is now dependent on you, the readers of PSPR, to engage with these ideas. To move us collectively toward a truly global science of social and personality psychology, we hope that Majority World and Western scholars and their institutions will learn from this diverse set of theoretical and conceptual perspectives and consider the ways in which they might impact their own scholarship, going forward.

## Notes

1. While the acronym “WEIRD” to describe “Western, Educated, Industrialized, Rich, Democratic” countries is more commonly used, we have chosen not to use it in this issue. The paper this acronym came from was highly generative in bringing attention to an important problem, but the acronym is now widely misused to describe entire countries, ignoring the diversity within them. Furthermore, the now-common practice of referring to people or countries as “non-WEIRD” (which was not done in the original article) implies that they are uneducated and undemocratic, and this is both inaccurate and hurtful.
